# Rate and Cause of Sudden Cardiac Death in the Young During the COVID-19 Pandemic and Vaccination

**DOI:** 10.1161/CIRCULATIONAHA.123.066270

**Published:** 2023-12-18

**Authors:** Monica De Gaspari, Ugo Fedeli, Mario Saia, Elisa Carturan, Kalliopi Pilichou, Domenico Corrado, Gaetano Thiene, Stefania Rizzo, Cristina Basso

**Affiliations:** 1 Department of Cardiac, Thoracic, Vascular Sciences and Public Health, University of Padua - Azienda Ospedaliera, Padova, Italy (M.D.G., E.C., K.P., D.C., G.T., S.R.).; 2 Epidemiological Department, Azienda Zero, Veneto Region, Italy (U.F.).; 3 Clinical Governance Unit, Azienda Zero, Veneto Region, Italy (M.S.).

**Keywords:** epidemiology, etiology, sudden cardiac death

Cardiovascular complications have been identified as consequences of COVID-19 infection and COVID-19 vaccines.^1,2^ Sun et al^3^ showed an increase of >25% in cardiac arrest/acute coronary syndrome emergency calls in the 16- to 39-year-old population during January to May 2021, and they found a significant association with the rates of first and second vaccine doses. A statewide analysis of out-of-hospital cardiac arrest in young people in Australia did not demonstrate increased rates of overall out-of-hospital cardiac arrest, myocarditis causing out-of-hospital cardiac arrest, or unascertained out-of-hospital cardiac arrest during the COVID-19 pandemic or after the introduction of COVID-19 vaccines.^4^ These contrasting findings and mass media claims raised concerns about vaccine-induced cardiovascular side effects. Our aim was to compare sudden cardiac death (SCD) rates and SCD attributable to myocarditis in young people across defined time periods before and during the pandemic and in the vaccination period.

Mortality records of residents 1 to 40 years of age in the Veneto Region, northeast Italy, between January 2018 and December 2022 were collected at the Regional Epidemiological Center, preceding the COVID-19 pandemic, at the onset of pandemic before vaccine availability, and during both the pandemic and vaccine availability. Yearly mortality in 2020, 2021, and 2022 was plotted against mortality in 2018 and 2019, overall and by causes of death selected according to the *International Classification of Diseases, 10th Revision*. All the cases referred from autopsies of SCD occurring in people 1 to 40 years of age were studied according to a standardized protocol. Sudden deaths are classified as SCD when the autopsy identifies a cardiovascular disease as the underlying substrate. When a cause of death is not found at autopsy including toxicology, death may be assumed to be SCD with normal heart.^5^ Data that support the findings of this study are available from the corresponding author on reasonable request. Analysis of mortality records is a mandatory activity of the Epidemiological Center; no Institutional Review Board approval and consent are needed.

The Veneto Region population 1 to 40 years ranged from 1 948 263 in 2018 to 1 868 987 in 2022. By December 2021, at least 1 vaccine dose was administered to 6% of people 5 to 11 years, 77% of those 12 to 19 years of age, and 84% of those 20 to 40 years of age.

According to death certificates, 103 subjects (80% male; mean age, 29 years) had SCD/other sudden death or cardiac arrhythmia/unspecified death (*International Classification of Diseases, 10th Revision* codes I44–I49, R96, R99). No variation was seen in the yearly rates (number of cases ×100 000 population: 2018–2019, 1.1; 2020, 0.9; 2021, 1.2; 2022, 1.1; 2021 versus 2018–2019, *P*=0.654). Figure (A) shows no change in mortality also if all circulatory and ill-defined deaths were examined (codes I00–I99, R00–R99). A sensitivity analysis was carried out that included all deaths from circulatory diseases, congenital malformations of the circulatory system, ill-defined causes, epilepsy, pulmonary edema/unspecified respiratory failure, and anoxic brain damage (codes I00–I99, R00–R99, G40–G41, G931, J81, J960, J969, Q20–Q28). Again, among the selected 366 deaths (75% male; mean age, 29 years), no variation was seen across study years. A decrease in overall mortality in 2020 is explained mainly by a decrease in traumatic/external causes of death (V01–Y98) with the first national lockdown on March 9, 2020.

**Figure.**
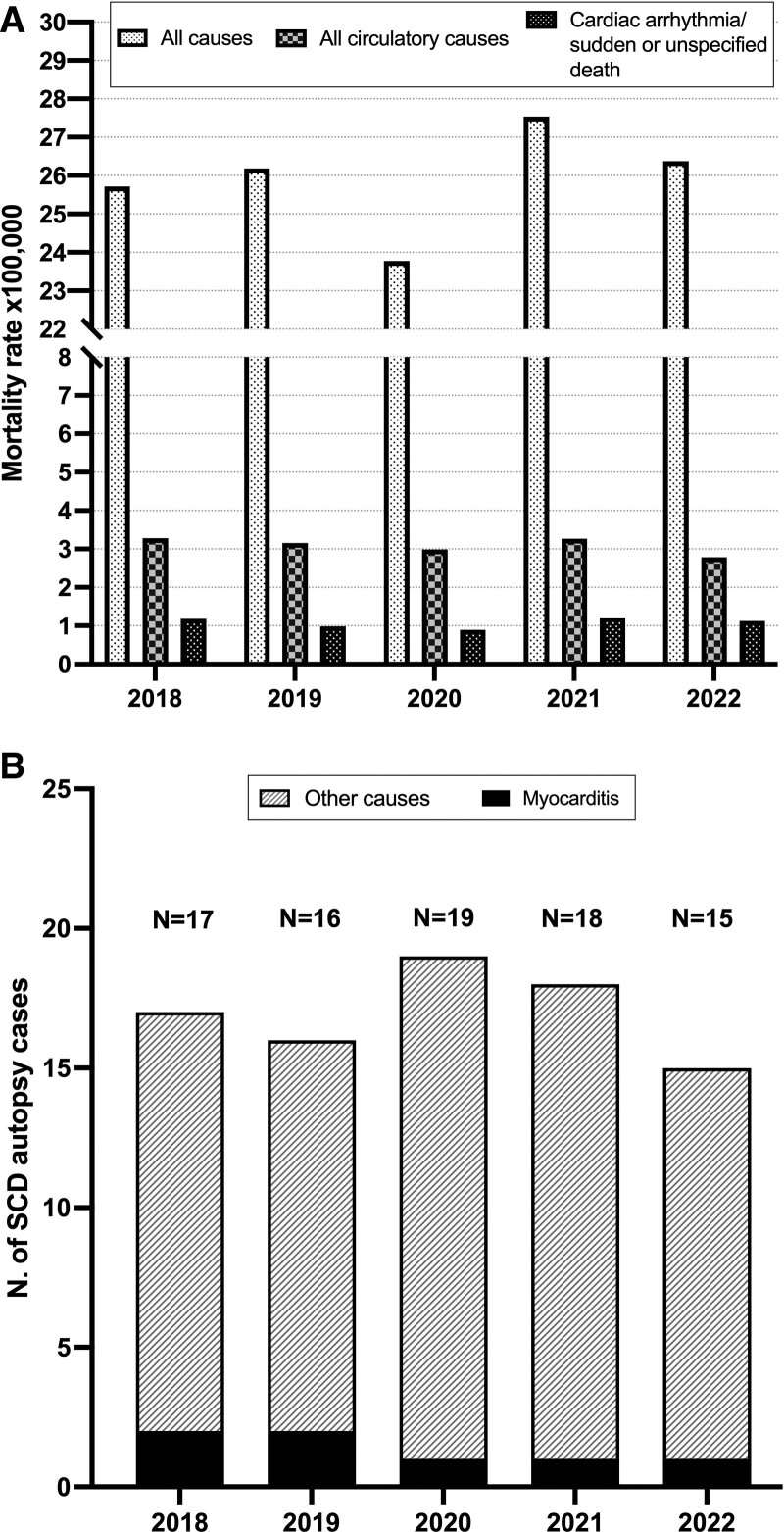
**SCD rates in the Veneto Region from 2018 to 2022. A**, Mortality records of residents 1 to 40 years in the Veneto Region (northeastern Italy) between January 2018 and December 2022 as collected at the Regional Epidemiological Center. Overall mortality, all circulatory/ill-defined causes (*International Classification of Diseases, 10th Revision* codes I00–I99, R00–R99), and sudden cardiac death (SCD)/other sudden death or cardiac arrhythmia/unspecified death (I44–I49, R96, R99) are shown. **B**, Total number of autopsies per year of young people 1 to 40 years of age residing in the Veneto Region who died between January 1, 2018, and December 2022 (n=85). In every column, the prevalence of myocarditis per each year is indicated.

In the same time interval, autopsy investigation revealed 85 SCD cases (age range, 1–40 years; mean age, 26.4±10.16 years; 63 male, 75%; 2018, n=17; 2019, n=16; 2020, n=19; 2021, n=18; 2022, n=15). Underlying diseases included coronary atherosclerosis (14), arrhythmogenic (10) and hypertrophic cardiomyopathy (6), aortic dissection (7), myocarditis (7), congenital malformations (3), mitral valve prolapse (2), and cerebral hemorrhage (1). The remaining 35 cases showed a normal heart, with a history of epilepsy in 5 and antipsychotic drugs and Brugada syndrome 1 each. Across the study period, myocarditis accounted for 11.76% in 2018, 12.50% in 2019, 5.26% in 2020, 5.56% in 2021, and 6.67% in 2022 (Figure [B]). Pericarditis and arteritis were never observed.

Twenty people, 8 in 2021 and 12 in 2022, had COVID-19 vaccination (15 BNT162b2 and 5 mRNA-1273). No inflammatory cause of death was found. Eight of them presented with SCD within 30 days of their COVID-19 vaccination (6 BNT162b2 and 2 mRNA-1273), 2 of them within 1 week. Causes of death were arrhythmogenic cardiomyopathy in 2 and hypertrophic cardiomyopathy and obstructive coronary atherosclerosis 1 each; 4 had unexplained SCD. Previous SARS-CoV-2 positivity was reported in only 8 (2 cases in 2021 and 6 in 2022; time interval, 1–296 days; median, 179 days). They were all vaccinated people. In 5 of them, COVID-19 positivity was ascertained after vaccination.

In conclusion, our analysis did not demonstrate increased rates of SCD in young people both during the pandemic and after the introduction of COVID-19 vaccination. Causes of SCD in young people, including those who experienced SCD within 30 days of their COVID-19 vaccination, were consistent with prepandemic causes as established by rigorous autopsy, and no increase in the prevalence of myocarditis has been observed.

## ARTICLE INFORMATION

### Sources of Funding

Drs De Gaspari, Rizzo, and Basso are supported by the Registry for Cardio-Cerebro-Vascular Pathology, Veneto Region, Italy (DGR No. 151 24/02/2023) and RF-2016-02363774 (DGR No. 735 28/05/2018), Ministry of Health, Rome, Italy. Dr Basso is supported by University of Padua Project BIRD221813.

### Disclosures

None.
